# Detection of anatomical variation during left internal jugular vein cannulation under ultrasound: A case report

**DOI:** 10.1097/MD.0000000000021129

**Published:** 2020-07-02

**Authors:** Hobum Cho, Geontae Kim, Sanghoon Song, Jaehwa Yoo, Mungyu Kim, Jiwon Chung, Sangho Kim, Sunyoung Park

**Affiliations:** Department of Anesthesiology and Pain Medicine, Soonchunhyang University Hospital Seoul, Seoul, Korea.

**Keywords:** anatomical variation, internal jugular vein, left side of neck

## Abstract

**Rationale:**

The left internal jugular vein has a higher possibility of anatomical variation than the right side. Therefore, the complication risk during cannulation is expected to be higher.

**Patient concerns:**

A 74-year-old woman was scheduled for elective surgery for left upper lobe wedge resection. We observed an anatomical abnormality at the location of the common carotid artery (CCA) and left internal jugular vein (IJV).

**Diagnosis:**

During the ultrasound, the left IJV was detected at the medial side of the CCA, and this anatomical variation was confirmed by color Doppler ultrasonography. Enhanced chest computed tomography showed that the left CCA ran across the left IJV from medial to lateral at the level of the clavicle.

**Intervention:**

A triple-lumen central venous catheter was inserted at the right IJV to avoid complications caused by the anatomical variation.

**Outcomes:**

There were no intraoperative or postoperative complications.

**Lessons:**

Anesthesiologists should consider anatomical variation during central venous cannulation, especially with the left IJV approach. Because of anatomical variation, ultrasound-guided intervention is highly recommended to prevent procedure-related complications.

## 1 Introduction

Central venous cannulation is a common and important procedure for volume resuscitation, drug and blood product administration, and hemodynamic monitoring. Although the placement of a central venous catheter is a common practice in clinical fields, it is an invasive procedure and has the possibility of complications, including bleeding, hematoma, malpositioning of the catheter, arrhythmia, infection, pneumothorax, and hemothorax.^[1]^

Among the various efforts to prevent these procedure-related complications, the use of ultrasound has been suggested because of a reduction in the complication rate and improved first-pass success when placing catheters in the internal jugular vein (IJV).^[2,3]^ Furthermore, cost-effectiveness for ultrasound-guided cannulation has been described.^[4–6]^ However, there is difficulty in using ultrasound during every procedure because of the limited number of devices for guiding procedures in every operating room at the same time. In such cases, central venous cannulation might be performed using the anatomical landmark-guided technique, and consequently mechanical complications such as carotid artery puncture, hematoma, and pneumothorax could be increased.^[7]^

We present a case with a tricky anatomical variation in the location of the left IJV identified during central venous cannulation under ultrasound guidance.

## 2 Case report

A 74-year-old woman (weight, 55 kg; height, 147 cm), classified as American Society of Anesthesiologists physical status 2, was admitted due to hemoptysis. She had a history of major depressive disorder and dementia. She was diagnosed with aspergilloma in the left upper lobe after bronchoscopic biopsy and was scheduled for elective surgery for left upper lobe wedge resection.

Upon arrival in the operating theater with premedication using glycopyrrolate (0.005 mg/kg, intramuscularly), standard monitoring devices, including an electrocardiogram machine, pulse oximeter, and oscillometric noninvasive blood pressure cuff, were applied. After the infusion of intravenous lidocaine (0.5 mg/kg), general anesthesia was induced and maintained using total intravenous anesthesia with propofol and remifentanil via effect site targeting using a target-controlled infusion system (Orchestra Primea; Fresenius Kabi AG, Bad Homburg, Germany). Propofol was monitored with the Schnider pharmacokinetic model and remifentanil with the Minto model. The target concentrations of propofol and remifentanil were 4 μg/mL and 6 ng/mL during induction and were maintained at 3 to 4 μg/mL and 1 to 6 ng/mL, respectively, according to hemodynamic changes. After loss of the eyelash reflex, a bolus dose of rocuronium (0.6 mg/kg) was administered; tracheal intubation was performed with a 37-Fr left-sided double-lumen tube (Mallinckrodt, Covidien, Ireland) using a McGrath videoscope (Aircraft Medical Ltd, Edinburgh, UK). The insertion depth was 26 cm from the upper incisors.

After confirming the modified Allen test, an arterial cannula was inserted in the right radial artery for continuous arterial blood pressure monitoring and blood sampling. Subsequently, we tried to insert a triple-lumen central venous catheter (ARROW Gard Blue; Arrow International, Reading, PA) in the left IJV under ultrasonographic guidance using the Seldinger technique, under sterile conditions and with the patient in the Trendelenburg position.

**Figure 1:**
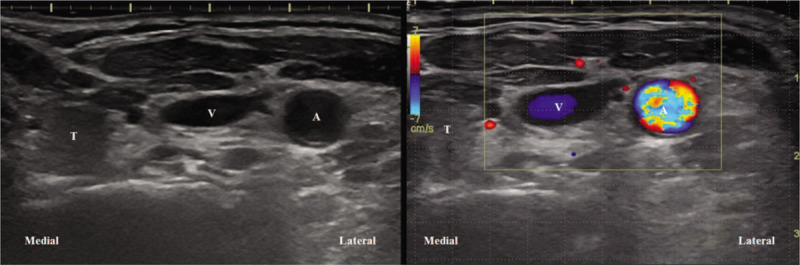
Ultrasonographic findings of anatomical variation in the internal jugular vein (IJV). The IJV was located on the medial side of the common carotid artery (CCA). Color Doppler ultrasonography confirmed CCA pulsation on the lateral side of the IJV. T: thyroid gland, A: CCA, V: IJV.

During ultrasonography, we observed an anatomical abnormality at the location of the common carotid artery (CCA) and IJV (Fig. 1). Generally, anatomical structures are visible by ultrasonography in the order of thyroid gland, CCA, IJV from the medial to lateral aspect. However, in our case, the IJV was on the medial side of the CCA, and this variation was confirmed by color Doppler ultrasonography (Fig. 1).

**Figure 2:**
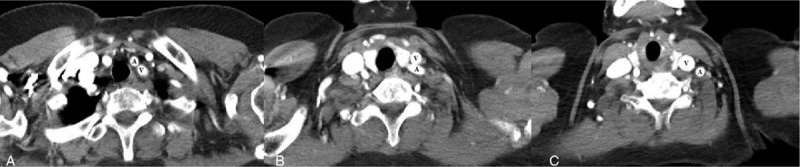
Computed tomographic findings of anatomical variation in the IJV. Serial-enhanced chest computed tomographic findings showed switching of the locations of the IJV and CCA at the level of the clavicle. A: CCA, V: IJV. CCA = common carotid artery, IJV = internal jugular vein.

Therefore, a triple-lumen central venous catheter was inserted through the right IJV to avoid complications due to the anatomical variation. We retrospectively reviewed a chest computed tomography (CT) image and found that the left CCA ran across the left IJV from the medial to lateral direction at the level of the clavicle (Fig. 2). The total anesthesia time was 280 minutes, and no intraoperative or postoperative complications occurred. The patient provided informed consent for publication of the case.

## 3 Discussion

Among central venous cannulation routes, the right IJV is preferred because of its larger size, more superficial position, and direct route to the heart.^[8]^ The risk of pneumothorax is also lower because the pleural dome is lower on the right side. Furthermore, there is little chance of damage to the thoracic duct on the right side.^[9]^ Therefore, the left IJV is selected when right IJV cannulation is unsuccessful or contraindicated, such as in the presence of significant right carotid artery stenosis, right neck dissection, or pre-existing right IJV thrombosis.^[10]^ However, we chose the left IJV as the central venous cannulation route at first, considering that the surgical site was the left lung. If a mechanical complication like pneumothorax occurred during cannulation of the left IVJ, it would be easy to repair directly during surgery.

Several previous studies described anatomical variations in IJV location. In the right neck, an IJV located on the medial side of the CCA has not been reported. In contrast, in the left neck, medial side locations have been reported (0%–2% of the total^[11–16]^) and the vessel size was smaller with a higher degree of overlap between the CCA and IJV.^[13,14,16]^ Furthermore, head rotation during central venous cannulation could increase the rate of overlap or medial side location of the IJV.^[15–17]^ Because of anatomical variation, ultrasound-guided intervention is highly recommended for cannulation of the left IJV to prevent procedure-related complications.

There were some risk factors causing mechanical complications during our procedure. First, we chose the left IJV for vascular access at first, considering that the surgical site was the left lung. Second, the overlap of the left IJV might have been aggravated because the patient's head was rotated to the opposite side for the procedure. In our case, anatomical variation in the left IJV was confirmed by chest CT, which was performed in a neutral position. However, anesthesiologists should keep in mind that head rotation itself could increase the complication risk. Finally, the patient was elderly. Troianos et al^[18]^ described an association between advanced age and predisposition to overlap between the IJV and CCA. Cervical arterial tortuosities increase related to old age. In such cases, we can avoid complications like arterial puncture or failure by confirming anatomical variations by ultrasonography.

In conclusion, anesthesiologists should consider anatomical variation during central venous cannulation, especially with the left IJV approach. If the left IJV must be accessed, ultrasound guidance is necessary to avoid complications, particularly in elderly patients.

## Author contributions

**Conceptualization:** Sanghoon Song, Jiwon Chung.

**Supervision:** Sun Young Park.

**Writing – original draft:** Ho Bum Cho, Geontae Kim.

**Writing – review & editing:** Ho Bum Cho, Jaehwa Yoo, Mungyu Kim, Sangho Kim, Sun Young Park.
